# Proteogenomic Characterization of Novel x-Type High Molecular Weight Glutenin Subunit 1Ax1.1

**DOI:** 10.3390/ijms14035650

**Published:** 2013-03-11

**Authors:** Miguel Ribeiro, Emmanuelle Bancel, Annie Faye, Mireille Dardevet, Catherine Ravel, Gérard Branlard, Gilberto Igrejas

**Affiliations:** 1 Department of Genetics and Biotechnology, University of Trás-os-Montes and Alto Douro, 5001-801 Vila Real, Portugal; E-Mail: jmribeiro@utad.pt; 2 Institute for Biotechnology and Bioengineering, Centre of Genomics and Biotechnology, University of Trás-os-Montes and Alto Douro, 5001-801 Vila Real, Portugal; 3 INRA UMR1095 UBP Génétique Diversité et Ecophysiologie des Céréales, 63039 Clermont-Ferrand, France; E-Mails: emmanuelle.bancel@clermont.inra.fr (E.B.); afaye@clermont.inra.fr (A.F.); mireille.dardevet@clermont.inra.fr (M.D.); catherine.ravel@clermont.inra.fr (C.R.); gerard.branlard@clermont.inra.fr (G.B.)

**Keywords:** *Triticum aestivum* L., wheat, storage proteins, glutenins, HMW-GS, *Glu-A1*, 1Ax1.1, MALDI-TOF-MS

## Abstract

Analysis of Portuguese wheat (*Triticum aestivum* L.) landrace ‘Barbela’ revealed the existence of a new x-type high molecular weight-glutenin subunit (HMW-GS) encoded at the *Glu-A1* locus, which we named 1Ax1.1. Using one-dimensional and two-dimensional electrophoresis and mass spectrometry, we compared subunit 1Ax1.1 with other subunits encoded at the *Glu-A1* locus. Subunit 1Ax1.1 has a theoretical molecular weight of 93,648 Da (or 91,508 Da for the mature protein) and an isoelectric point (pI) of about 5.7, making it the largest and most acidic HMW-GS known to be encoded at *Glu-A1*. Specific primers were designed to amplify and sequence 2601 bp of the *Glu-A1* locus from the ‘Barbela 28’ wheat genome. A very high level of identity was found between the sequence encoding 1Ax1.1 and those encoding other alleles of the locus. The major difference found was an insertion of 36 amino acids in the central repetitive domain.

## 1. Introduction

Wheat is one of the most widely grown crops in the world and a major source of protein in the human diet. Roughly 85% of the total protein contained within wheat endosperm is gluten, a very large complex responsible for the unique viscoelastic properties of wheat dough [1,2]. The major components of gluten are the monomeric gliadins and the polymeric glutenins. Glutenins mainly confer dough elasticity and gliadins dough extensibility essential for bread-making [3,4]. Glutenins are classed as high molecular weight-glutenin subunits (HMW-GS) encoded at *Glu-1* loci and low molecular weight-glutenin subunits (LMW-GS) encoded at *Glu-3* loci. HMW-GS are further subdivided into high *M*_r_ x-type and low *M*_r_ y-type subunits. Tightly linked pairs of genes encoding one x-type and one y-type subunit are found on the 1A, 1B and 1D chromosomes of hexaploid bread wheat [5].

Both x- and y-type HMW-GS have a large central elastomeric repetitive domain flanked by short non-repetitive *N*- and *C*-terminal domains. According to results from circular dichroism (CD) spectroscopy and computer prediction from amino acid sequences, the *N*- and *C*-terminal domains are probably both rich in α-helices [6] and the dominant structural feature of the central repetitive domain is the presence of β-reverse turns [7]. The main difference between the two types of HMW-GS is that 18 residues of the *N*-terminal domain after residue 33 are deleted in x-type subunits, such that the domain is 81 to 89 residues long in x-type subunits but 104 residues long in y-type. In contrast, the *C*-terminal domain of both types is made up of 42 residues [8].

The distribution and number of cysteine residues in all three domains are of particular interest because disulfide bonds alter the polymeric structure and the conformation of the protein, which in turn is critical for the viscoelastic properties of the wheat dough [9,10]. All y-type subunits have five cysteines in their *N*-terminal domain. Two of these residues are localized in the 18-residue sequence that is deleted in x-type subunits, which therefore have no more than three cysteines in the *N*-terminal domain. HMW-GS *N*-terminal domains therefore have one (in 1Bx14 and 1Bx20), three (in 1Bx7, 1Bx17, all 1Dx and 1Ax subunits) or 5 (all y-type subunits) cysteine residues. All HMW-GS have only one cysteine residue in the *C*-terminal domain.

Cysteine residues are only present within the central repetitive domains in y-type subunits, but are not usually present in the equivalent domain in x-type subunits, although there are a few exceptions. Like y-type subunits, the central repetitive domain of the 1Dx5 subunit contains an additional cysteine residue. However, it is in a different position than cysteines in y-type subunits, being in the first repetitive block adjacent to the *N*-terminal domain [11]. Buonocore *et al.*[10] suggested that this “extra” cysteine residue might be the major factor explaining why this gluten subunit is associated with good quality. In the 1Ax2*^B^ subunit, an x-type HMW-GS encoded at the Glu-A1 locus, there is a cysteine residue in the central domain in a different position compared to that in 1Dx5 [12].

As well as cysteine residues, the specific amino acid composition of HMW-GS is important in determining their elastic and polymeric behavior and thus the rheology and processing quality of wheat dough [13]. For example, the repetitive sequences rich in Gln residues form an extensive interchain array of hydrogen bonds, which makes wheat dough more elastic [14]. Tyrosine cross-links also influence the rheological properties of dough and gluten [15].

In general, the HMW-GS are the major genotypic determinants of dough strength and determine the suitability of the wheat for bread-making by conferring dough viscoelasticity [16]. The variability among the HMW-GS alleles has been repeatedly shown to influence bread-making quality [17]. The analysis of genetic variability of HMW-GS was initiated in the 1980s by Payne and Lawrence [18] when 20 HMW-GS alleles were identified by electrophoresis of glutenin proteins on acrylamide gels, namely 3 alleles at the *Glu-A1* locus, 11 at the *Glu-B1* locus and 6 at the *Glu-D1* locus. Several other HMW glutenin alleles were later identified [16,19–23].

Analysis of the genetic diversity of ‘Barbela’, a bread wheat population cultivated in Portugal, revealed the existence of a new HMW-GS, named 1.1, encoded at the *Glu-A1* locus. We previously found that this specific subunit is implicated in the high dough extensibility of flour from some ‘Barbela’ wheat lines [21], with alveograph L values up to 224 mm (G. Igrejas, unpublished data). This contrasts with other data showing that the 1Ax1.1 allelic effect on dough extensibility is similar to the effect of the 1Ax1 subunit, and suggests that the rheological data may be influenced by the environment in which experiments are carried out. However, there are also data from a series of crosses between ‘Barbela’ wheat lines encoding the subunit 1.1 and other bread wheat varieties showing that different HMW-GS combinations influence wheat quality differently [24].

Here we report the sequence of a new x-type HMW-GS encoded at the *Glu-A1* locus and the impact of specific differences in the central repetitive domain on the technological value of the resulting wheat flour.

## 2. Results and Discussion

**Figure 1:**
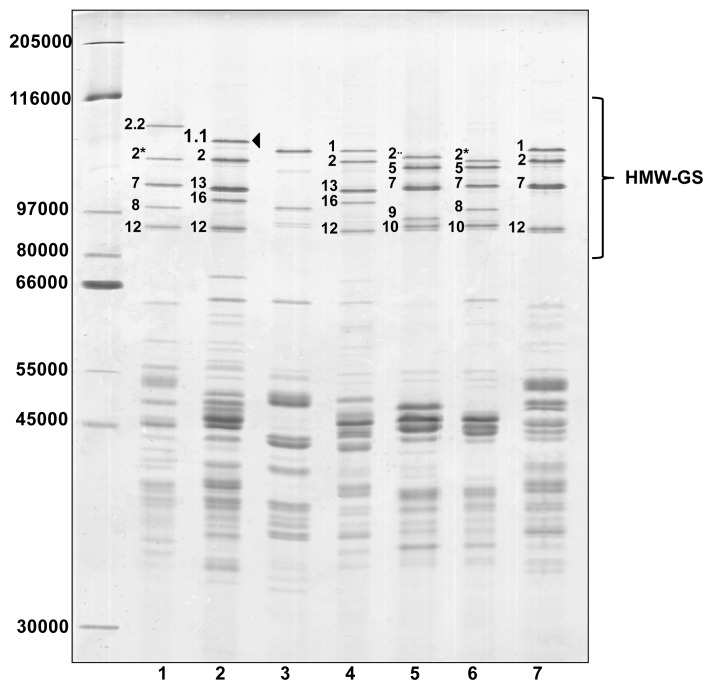
Reduced and alkylated glutenin subunit patterns of selected bread wheat accessions analyzed by SDS–PAGE using a 12% gel for both high molecular weight-glutenin subunits (HMW-GS) and low molecular weight-glutenin subunits (LMW-GS). The HMW-GS are labeled according to the nomenclature of Payne and Lawrence [18]. Lane 1, ‘Fukihokomugi’; lane 2, ‘Barbela 28’; lane 3, ‘PI 355’; lane 4, ‘Carala’; lane 5, ‘211 12 04’; lane 6, ‘13-21’; lane 7, ‘Konini’. The arrowhead points to the *Glu-A1* allele 1.1. Sizes (in daltons) of protein molecular weight markers are shown on the left.

Glutenin subunits of the ‘Barbela 28’ wheat line were compared to those from other wheat lines and varieties with identical alleles encoded at *Glu-B1* and *Glu-D1* loci by sodium dodecyl sulfate-polyacrylamide gel electrophoresis (SDS-PAGE), shown in Figure 1.

The wheat line ‘PI 355’ was kindly provided by Q. Y. Li who reported the presence of subunit 1.1 in *Triticum turgidum* ssp. *Dicoccum*[25]. There is no band in the profile of the ‘PI 355’ sample with the same electrophoretic mobility as the 1.1 subunit found in ‘Barbela 28’. The band with the least mobility in the ‘PI 355’ line has an apparent molecular weight very similar to subunit 1 found in ‘Carala’ and ‘Konini’. To avoid confusion, it should be noted that the HMW-GS named 1.1 encoded at the *Glu-A1* locus was described first in an analysis of genetic diversity of wheat grown in Portugal [21]. As expected the ‘211 12 04’ wheat line shows an unusual x-type allele encoded at the *Glu-A1* locus, named Ax2·· [26]. The SDS-PAGE shows that HMW-GS 1.1 is less mobile than HMW-GS 1, with an apparent molecular weight between those of subunits 1Dx2.2 and 1Ax1.

**Figure 2:**
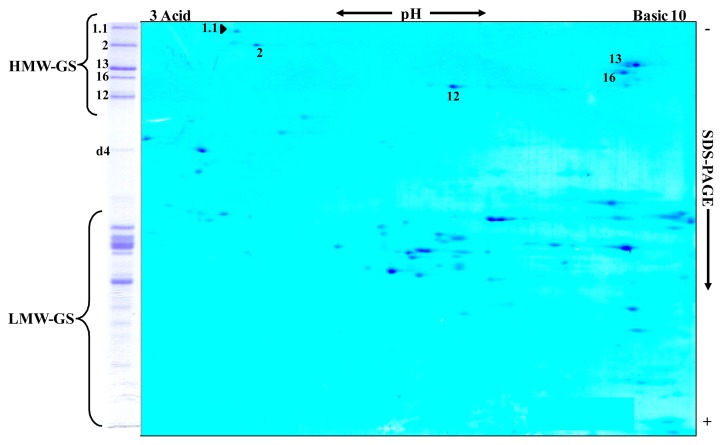
Two-dimensional electrophoresis pattern (IEF × SDS-PAGE) of the HMW-GS of wheat line ‘Barbela 28’. The arrowhead points to the *Glu-A1* allele 1.1. One-dimensional SDS-PAGE separations are shown to the left of the two-dimensional separations.

The HMW-GS detected by SDS-PAGE were further identified by two-dimensional electrophoresis (2-DE), *i.e.*, isoelectric focusing (IEF) followed by SDS-PAGE. The 2-DE profile of the ‘Barbela 28’ wheat line is showed in Figure 2.

All subunits analyzed were distinctly separated and most formed a single spot on the 2-DE gel. Comparison with other 2-DE patterns, and in particular with the ‘Carala’ pattern, revealed that subunit 1.1 in ‘Barbela 28’ was clearly distinguishable from subunit 1.

**Figure 3:**
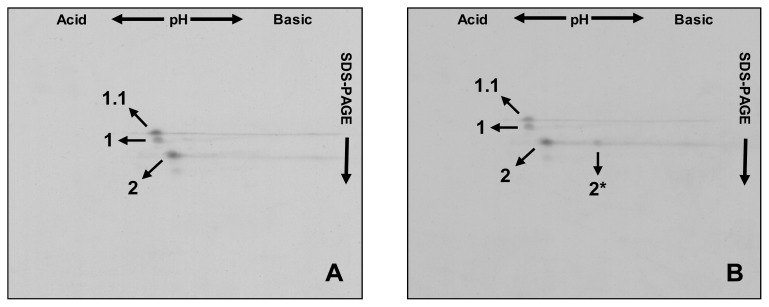
Overlap of two-dimensional patterns of HMW-GS from ‘Barbela 28’ wheat line and other extracts (**A**) with the variety ‘Carala’ and (**B**) with an equal mixture from varieties ‘Carala’ and ‘Atlas 66’. Subunits 1.1, 1, 2 and 2* are indicated.

The following 2-DE patterns aid in distinguishing between different *Glu-A1* encoded alleles. First the line ‘Barbela 28’ is compared with the variety ‘Carala’ (Figure 3A) then with a 1:1 mixture of glutenin extracts from ‘Carala’ and ‘Atlas 66’ (Figure 3B).

Subunit 1.1 is clearly distinguished from subunit 1, being slightly more acidic with a higher molecular weight. Subunit 2* had a two-dimensional profile very different from subunits 1.1 and 1, being more basic with a lower molecular weight. Subunit 2, present in each variety, overlapped precisely in all the 2-DE profiles.

**Figure 4:**
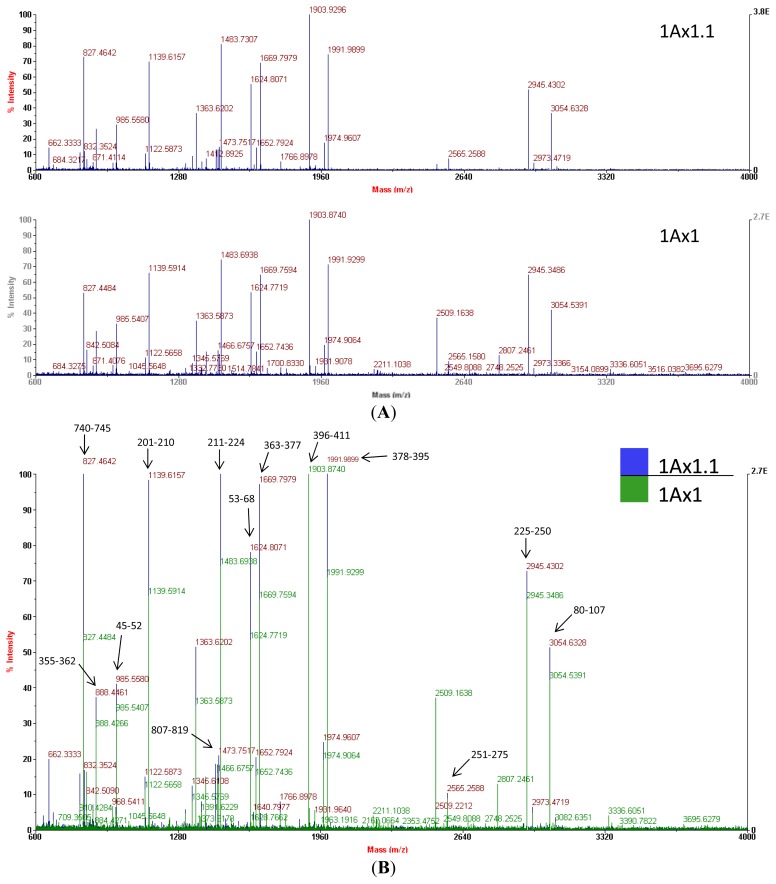
MALDI-TOF mass spectra of the two high molecular weight glutenin subunits 1Ax1 and 1Ax1.1. (**A**) Individual mass traces and (**B**) Overlaid mass traces.

Mass spectrometry of trypchymo-digested peptides extracted from gel spots showed that subunit 1.1 (from the ‘Barbela’ wheat variety) and subunit 1 (from the ‘Carala’ wheat variety) are very similar (Figure 4). The latter subunit is strongly associated with good bread-making quality [27]. Subunit 1.1 peptide masses were used to search an NCBI non-redundant protein sequence database using the MASCOT tool and showed the greatest resemblance to subunit 1 (GenBank^®^ accession number CAA43331.1). Of the 39 subunit 1.1 peptide masses, 20 values matched those of subunit 1 giving an identity score of 184 (with 27% of sequence coverage). In Figure 4B, a few MH+ ion peaks, like one at *m*/*z* 983.5233, are not shown because of the scale (34–41). Mass spectra were used at this stage to confirm that the isolated protein was indeed an x-type high molecular weight glutenin subunit encoded at the *Glu-A1* locus. As *N*- and *C*-terminal domains of the subunits 1Ax1.1 and 1Ax1 are very similar, data from mass spectra and from sequence databases were used together to design suitable primers.

Specific primers were designed to amplify the *Glu-A1* locus from ‘Barbela 28’ genomic DNA, *i.e.*, the gene encoding HMW-GS 1.1, and the amplified DNA was sequenced and analyzed. The 1Ax1.1 ORF is 2601 bp long, which is slightly longer than the 2493-bp ORF reported for the gene that encodes subunit 1 [28] and is consistent with the higher molecular weight of subunit 1.1 found in gel electrophoresis (Figure 1).

**Figure 5:**
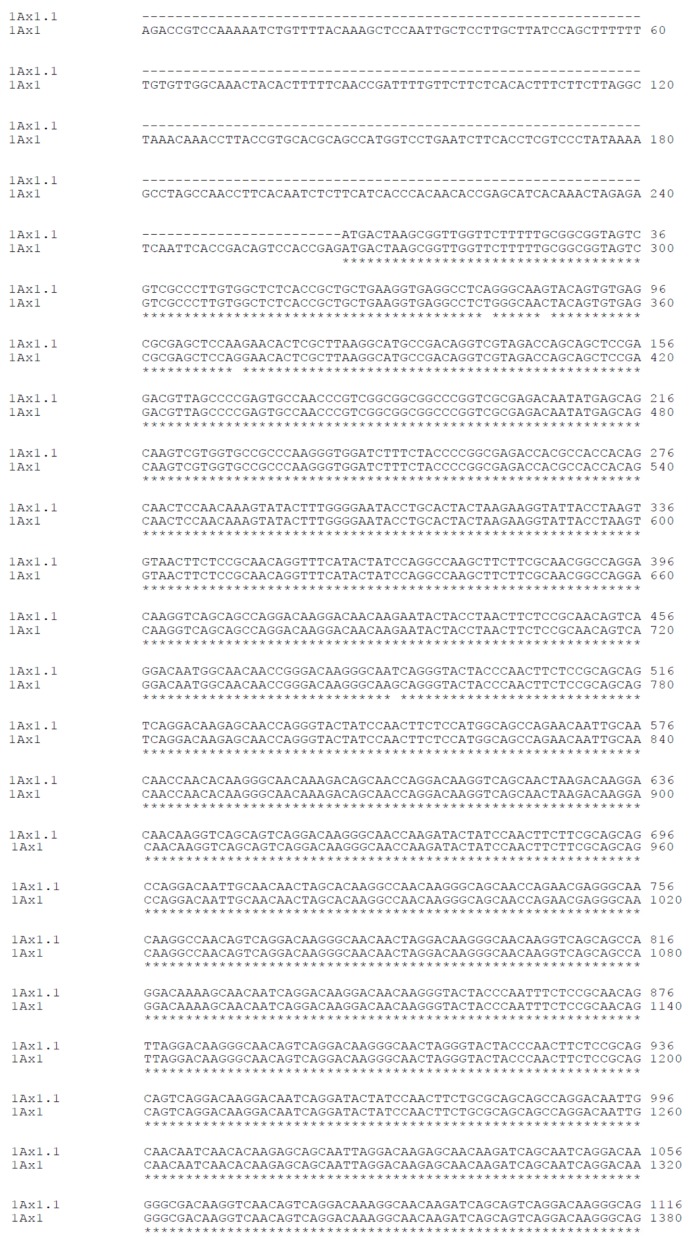

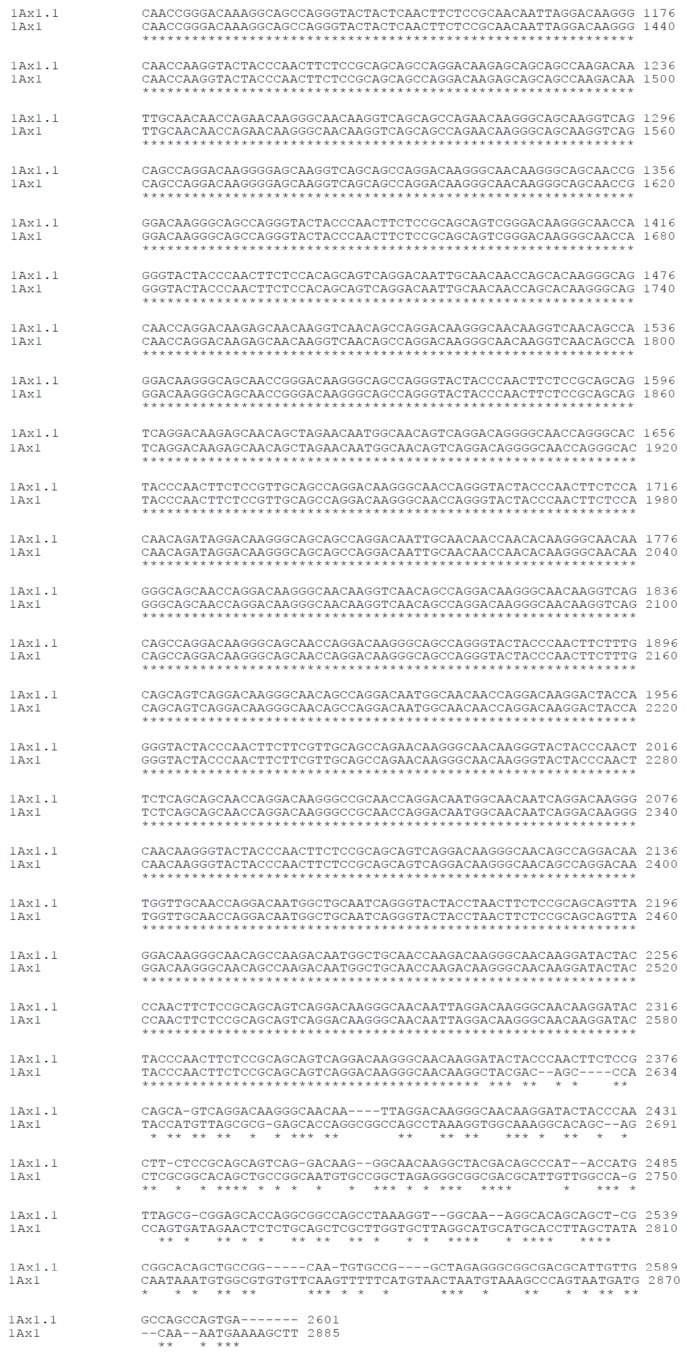
Alignment of 1Ax1.1 (GenBank^®^ accession no. JN172932.1) and 1Ax1 (GenBank® accession no. X61009.1) HMW-GS nucleotide coding sequences. The identity between the two sequences is 95%.

A high level of nucleotide identity (95%) was found between coding sequences of 1Ax1.1 and 1Ax1 HMW-GS genes (GenBank^®^ accession nos. JN172932.1 and X61009.1 respectively). The sequence alignment of the 1.1 and 1 subunit ORFs is shown in Figure 5. High levels of identity with other *Glu-A1* encoded alleles were also found, e.g., with the null allele and with 2··, 2* and 2*^B^ alleles (GenBank^®^ accession nos. HQ613179.1, DQ533690.1, M22208.2 and EF055262.1 respectively).

Several differences were found between the two sequences. As well as the 1.1 ORF having 108 more base pairs than the ORF of subunit 1, there are 46 single-base, 12 two-base, 9 three-base, 1 four-base and 1 six-base substitutions. There are also 1 single-base, 4 two-base and 1 four-base insertions and 6 single-base, 4 two-base, 2 four-base and 1 five-base deletions.

The HMW-GS 1.1 peptide sequence is 866 amino acids long, and HMW-GS 1 is 830 amino acids long. The predicted amino acid composition of *N*- and *C*-terminal domains of subunit 1.1 is identical to subunit 1, as expected from mass spectrometry data. All the subunit 1 cysteines were conserved in subunit 1.1 and no extra cysteine residue was found in the central repetitive domain of 1.1. This indicates that the patterns of intra- and inter-chain disulfide bonds are identical for both proteins.

**Figure 6:**
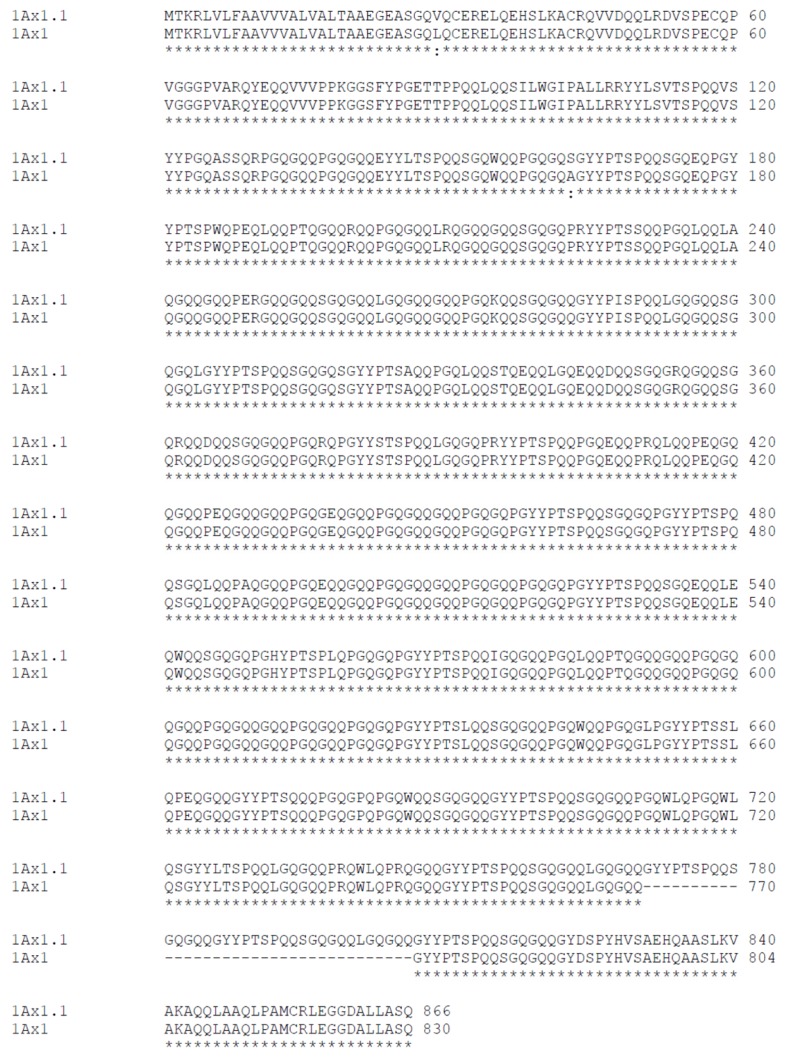
Alignment of the predicted primary structure of 1Ax1.1 and 1Ax1 HMW-GS. The dashes indicate the deletion of 36 amino acids at the end of the central repetitive domain at position +771 relative to the 1Ax1.1 sequence. Substitutions are indicated by colons.

In addition, two substitutions in highly conserved residues were found at positions +29 and +163 in the *N*-terminal and central repetitive domains, respectively (Figure 6).

In the central domain, there are some repeats based on combinations of motifs of tripeptides, like GQQ, hexapeptides, like PGQGQQ and PGQLQQ, and nonapeptides, like LRQGQQGQQ. The tripeptides also appear in tandem with hexapeptides, forming nine-residue motifs, like PGQGQQGQQ. Some nonapeptides are interspersed with hexapeptides, forming 15-residue motifs, like PGQGQQLRQGQQGQQ, while the hexapeptides occur in tandem arrays. The presence of numerous glutamine (Q) residues should be noted, as these amino acids are involved in determining the elastic properties of wheat dough.

The insertion of 36 amino acids at the end of the central repetitive domain at position +771 in 1Ax1.1 (Figure 6) leads to some changes in the repetitive motifs compared with HMW-GS 1, namely the tripeptide GQQ, hexapeptide LGQGQQ and nonapeptide GYYPTSPQQ.

Comparing the MS data to the peptide sequence, we conclude that it was not possible to distinguish the two sequences by MALDI PMF either for the matched sequences or the unmatched masses. This is because almost all the peptides likely originate from tryptic cleavages. Peptide masses corresponding to fragments with any of the three differences found in the subunit 1Ax1.1 peptide (the two substitutions and the 36-aa insertion; Figure 6) were not detected.

Similar results regarding sequence coverage were reported by Qian *et al.*[29], who also reported difficulties in discriminating between two HMW-GS encoded at the *Glu-A1* locus (1Ax1 and 1Ax2*). Previously, Hickman *et al.*[30] indicated that HMW subunits were not suitable for MS measurement because of poor resolution and even with individual proteins, the spectral peaks were broad. The relatively low frequencies of arginine and lysine residues in these proteins, which gives rise to mainly large tryptic peptides, and the close sequence similarity within this group of subunits, are consistently referred to as the main problems in characterizing HMW-GS by a MALDI PMF approach [29,31].

As reported elsewhere [29,31], we also attempted to measure the *M*_r_ of intact HMW-GS 1 and 1.1. The predicted masses of the intact subunits identified above, based on published DNA sequences, are in good agreement with the observed *m*/*z* values obtained by linear MALDI-TOF (Table 1). The difference was less than 0.5% for both subunits, which is not significant if we consider the mass of the HMW-GS under analysis. Moreover, if we compare our results with previous data estimating the *M*_r_ of HMW-GS, this value is sufficient to be able to discriminate between the two subunits.

Analyzing old varieties or landraces has led to the identification of unusual allelic variants, some of which are already being incorporated into the genomes of current commercial wheat. Here we aimed to identify and characterize this unusual subunit. Proteomics was used to clearly distinguish subunit 1.1 from other subunits encoded at the *Glu-A1* locus like subunits 1, 2, 2*. In 2-DE, HMW-GS 1.1 migrates as a slightly more acidic protein than HMW-GS 1, although based on predictions from the primary sequence, the pI of both subunits should theoretically be 5.7. This makes subunit 1.1 the most acidic HMW-GS encoded at *Glu-A1* locus reported so far (Figure 3).

The 1.1 ORF is predicted to encode a protein sequence with a *M*_r_ of 93,649 Da (or 91,508 Da for the mature protein), the highest *M*_r_ reported for an HMW-GS encoded by the *Glu-A1* locus. According to Don *et al.*[32], the very high molecular weight fraction of glutenins is considered important in determining wheat flour quality. HMW-GS are essential for the formation of glutenin macropolymer (GMP). Thus, the very high molecular weight of subunit 1.1 may have positive effects on the formation and size of the GMP. However, the presence or absence of the 1Ax1 subunit hardly affects the quantity of GMP present [32]. It is possible that subunit 1.1 has a similar or stronger positive effect on the formation of GMP. GMP and its rheological properties are widely reported to be good quality predictors [33–35]. Moreover, Weegels *et al.*[34] reported that GMP content is strongly related to the maximum resistance (R_max_) and extensibility of dough.

The insertion of 36 amino acids at the end of the central repetitive domain leads to some differences in the HMW-GS 1.1 repeat motifs compared with HMW-GS 1. The longer repetitive sequence may allow the formation of more hydrogen bonds and induce conformational differences. D’Ovidio *et al.*[36] suggested that the repetitive β-turn motifs in the central domain of HMW-GS explain their anomalously slow migration in SDS-PAGE. This might also explain the distinct difference in mobility of subunits 1Ax1.1 and 1Ax1, not only by the difference in mass (3.8 kDa difference in theoretical *M*_r_) but also by differences in the number of repetitive domains in the protein secondary structure.

Preliminary technological tests were conducted to identify potential differences in flour quality conferred by 1Ax1.1 and 1Ax1 HMW-GS. Common tests used to evaluate wheat technological quality showed no significant differences between 1Ax1.1 and 1Ax1 (Table 2). This result is consistent with the identical patterns of intra- and inter-chain disulfide bonds that both proteins presumably form.

The *Glu-A1* allelic comparisons were performed using different sister lines that had different *Glu-B1* alleles (either 7–8 or 13–16) yet were homogenous for *Glu-D1*, *Glu-A3*, *Glu-B3* and *Glu-D3* loci. No consistent differences were associated with subunit 1.1 or 1. However, significant environmental effects were associated to the location where wheat was grown, either France or Portugal. The highest values were found in France for the alveograph parameters P, L W, Ie, and for protein content and grain hardness.

D’Ovidio *et al.*[37] reported an unusually large insertion of 561 bp within the repetitive domain of a functional HMW glutenin gene encoded at *Glu-D1* locus. Later, He *et al.*[38] studied the relationship between the subunit size and the effect on dough mixing properties of flour made from wheat lines transformed with a gene encoding an extended form of subunit 1Dx5. They found that none of the transgenic lines expressing the extended form of the 1Dx5 subunit showed the “overstrong” mixing properties exhibited by transgenic lines expressing the wild type 1Dx5 transgene. Similarly, in this work we demonstrated that the larger size of the subunit 1Ax1.1 and modification of the repetitive motifs did not produce a significant alteration in the rheology of dough. However, additional tests, including bread-making tests, are needed with subunit 1Ax1.1 expressed in different genetic backgrounds to ascertain whether this new allele has a positive impact on the size of polymers formed in wheat flour.

## 3. Experimental Section

### 3.1. Plant Materials

The ‘Barbela’ wheat population used was from the germplasm bank of the University of Trás-os-Montes and Alto Douro (UTAD), Vila Real, Portugal. Line 28 of ‘Barbela’ wheat was used throughout the study. The other germplasm used came from the germplasm bank of Institut National de la Recherche Agronomique (INRA), Clermont-Ferrand, France. The varieties used and their respective *Glu-1* alleles are listed in Table 3.

Other wheat lines and varieties with the same alleles encoded at *Glu-B1* and *Glu-D1* loci as in ‘Barbela’ were used as controls. For the two-dimensional analysis, the varieties ‘Carala’ and ‘Atlas 66’ were selected as controls as they have the same composition as ‘Barbela’ at *Glu-B1* and *Glu-D1* loci.

For mass spectrometry analysis, the protein spots correspond to HMW-GS 1Ax1.1 from ‘Barbela 28’ and 1Ax1 from ‘Carala’.

For analysis of the *Glu-A1* gene, in addition to ‘Barbela 28’ genomic DNA, genomic DNA from *Triticum urartu* (A genome), *Triticum speltoides* (B genome) and *Triticum tauchii* (D genome), and of the *Triticum aestivum* L. ‘Chinese Spring’ variety was used to verify the specificity of amplification of the *Glu-A1* locus in the ‘Barbela 28’ wheat line.

The different ‘Barbela 28’ sister lines carrying either subunit 1.1 or subunit 1 with either *Glu-B1*-encoded subunit 13–16 or subunit 7–8 were grown in two locations (in Vila Real, Portugal and in Clermont-Ferrand, France) in the year 2010–2011. At least two sister lines each having identical HMW-GS and LMW-GS composition were grown in the two locations with conventional fertilization and full fungicide protection.

### 3.2. Technological Tests

Each sample of flour from a specific wheat genotype was analyzed in duplicate and in two different locations: Vila Real, Portugal and Clermont-Ferrand, France.

Grain was milled using a Cyclotec lab mill (Tecator) to produce wholemeal. Flour protein content and kernel hardness were estimated on a wholemeal sample by near infrared reflectance (NIR–Percon Inframatic 8620) according to AACC (1995) Approved Methods 39-11 and 39–70A, respectively.

The Alveograph parameters such as tenacity (P), extensibility (L), the deformation energy or dough strength (W), tenacity/extensibility ratio (P/L) and elasticity index (Ie) were determined according to the ICC standard N°121.

### 3.3. Electrophoresis

HMW-GS were extracted according to the sequential method of Singh *et al.*[39] with some modifications. The HMW-GS present in the pellet were reduced and alkylated in a 50% propanol solution with 1% dithiothreitol and 2.5% iodoacetamide, respectively. Glutenin subunits were separated in a resolving gel (12.52% T and 0.97% C). The gels were stained with Coomassie blue and alleles were identified by referring to known alleles in control genotypes. The nomenclature of the HMW-GS corresponds to the terminology used by Payne and Lawrence [18].

Two-dimensional electrophoresis (2-DE) of HMW-GS was carried out as previously described [40]. Briefly the HMW-GS fraction obtained was added to extraction solution containing 4% CHAPS, 7 M urea, 2 M thiourea, 1% IPG buffer, 20 mM DTT and milliQ ultrapure water. The flour plus the extraction solution were vortexed, sonicated and centrifuged. The rehydration of strips for isoelectric focusing (IEF, pH 3–10) was carried out using the rehydration solution consisting of extraction solution with bromophenol blue. IEF was carried out at 60 kVh followed by SDS-PAGE on gels (12.52% T, 0.97% C) that were then stained with Coomassie Blue.

### 3.4. Protein Identification by Mass Spectrometry (MS)

Selected spots were excised and treated with successive washes of ammonium bicarbonate (NH_4_HCO_3_) plus acetonitrile (ACN) in order to remove Coomassie Blue. To dehydrate the gel spots, they were incubated in 100% ACN then the ACN was completely removed.

For in-gel digestion at 37 °C overnight, 15 μL of 10 ng/μL trypsin (V5111; Promega, Madison, WI, USA) in 25mM NH_4_HCO_3_, 15 μL of 10 ng/μL chymotrypsin (C6423, Sigma, St. Louis, MO, USA) in 50 mM NH_4_HCO_3_, and 10 mM CaCl_2_ were added to the dry gel. Gel pieces were centrifuged and 10 μL ACN was added to extract the peptides. The mixture was sonicated for 5 min and centrifuged at 5000*g* for 5 min. For matrix-assisted laser-desorption/ionization time of flight mass spectrometry (MALDI-TOF-MS), 1 μL of supernatant was loaded directly onto the MALDI target. One microliter of the matrix solution (5 mg/mL α-cyano-4-hydroxycinnamic acid in 50% ACN/0.1% trifluoroacetic acid) was added immediately and the mixture was left to dry at room temperature.

Peptide mass fingerprinting was performed using a Voyager DE-Pro model of MALDI-TOF mass spectrometer (Perspective BioSystems, Farmingham, MA, USA) used in positive-ion reflector mode. External calibration was performed with a standard peptide solution (ProteomiX 3, LaserBio Labs, Sophia-Antipolis, France). Internal calibration was performed using peptides resulting from auto-digestion of trypsin. Mono-isotopic peptide masses were obtained using MALDI-TOF-MS analysis and were searched against the NCBI non-redundant protein sequence database using the MASCOT tool (Matrix Science). Matches to protein sequences from the *Viridiplantae* taxon were considered acceptable if a significant score was obtained from MASCOT, which rates scores as significant if they are above the 95% significance threshold (*p* < 0.05). Protein scores greater than 70 were considered significant.

The *M*_r_ of intact HMW-GS 1 and 1.1 was measured according to Liu *et al.*[41].

### 3.5. Analysis of the HMW-GS 1Ax1.1 Coding Sequence

DNA extraction was carried out according to Stein *et al.*[42]. PCR was performed in a reaction volume of 25 μL using 25 ng of genomic DNA, 1 U *Taq* DNA polymerase in 1× PCR buffer, 200 μM dNTP mix and 10 pmol of each primer. Primers for the HMW-GS 1Ax1.1 coding sequence were: forward 5′-CGAGATGACTAAGCGGTTGGTT-3′ and reverse 5′-GAGTTCTATCACTGGCTGGCCA-3′. The PCR reaction started with an initial cycle at 94 °C for 4 min, followed by 35 cycles of 94 °C for 1 min, 65 °C for 1 min and 72 °C for 2 min, then a final extension at 72 °C for 5 min.

PCR amplification products were analyzed by agarose gel electrophoresis in 1% agarose and stained with ethidium bromide. The 1Ax1.1 sequence obtained was deposited in GenBank^®^ under the accession number JN172932.1. Open reading frame (ORF) detection was carried out with the ORF Finder software from NCBI. The 1Ax1.1 sequences alignment was performed with ClustalW2 tool from EBI.

## 4. Conclusions

The study provides a better understanding of the genetic and molecular basis of the unique HMW-GS 1.1. Although no significant differences were found in some technological tests, this subunit potentially influences polymer size and hence bread-making quality. The ‘Barbela’ lines, including those bearing the 1.1 subunit, have already been associated with good biscuit quality [24].

Reporting a new x-type allele at the *Glu-A1* locus means that the genetic variability available for selection is increased. It is expected that through advanced technologies, such as proteomics, the identification of novel or unusual alleles from the large wheat gene pool will provide tools for breeders to further improve dough properties and gluten quality. Proteomics is a powerful tool to elucidate gluten protein expression, diversity and interactions. The resulting knowledge will allow a better conservation of wheat genetic resources. In the future, it would be interesting to extend our knowledge of this novel HMW-GS allele at the molecular level, its heritability (extensibility is a character with low heritability) and its role in determining end use value.

## Figures and Tables

**Table 1:** Comparison of HMW-GS molecular masses deduced from coding gene sequences with those determined by MALDI-TOF-MS.

HMW-GS	Deduced *M*_r_ from coding gene		*M*_r_ by MALDI-TOF-MS		Variation	
		
	Origin	Mass (Da)	Origin	Mass (Da)	Difference (Da)	Error (%)
1Ax1	‘Hope’	87,678	‘Carala’	87,859	−181	0.21
1Ax1.1	‘Barbela 28’	91,508	‘Barbela 28’	91,858	−350	0.38

**Table 2:** Analysis of the impact of the allelic variation on quality parameters in wheat lines containing either 1Ax1.1 or 1Ax1 HMW-GS. Quality parameters are kernel protein content, grain hardness, tenacity (P), extensibility (L), deformation energy (W), tenacity/extensibility ratio (P/L) and elasticity index (Ie).

Comparison of Means		Alveograph Parameters					Protein Content % dm	GrainHardness
	
Location	Genotype (*Glu-A1; Glu-B1*)	P mmH_2_0	L mm	W 10^−4^ J	P/L mmH20 mm^−1^	Ie		
Portugal	1; 7 + 8	44.8	101.3	80.3	0.5	25.1	15.0	53.7
	1.1; 7 + 8	51.7	110.7	114.4	0.5	27.6	14.2	35.6
	1; 13 + 16	63.8	143.4	184.0	0.4	35.2	14.3	23.0
	1.1; 13 + 16	57.8	119.6	141.1	0.5	30.9	15.3	41.9
France	1; 7 + 8	70.4	208.6	295.1	0.33	44.7	17.5	77.0
	1.1; 7 + 8	72.6	181.8	243.1	0.40	37.0	14.8	49.7
	1; 13 + 16	93.5	148.8	370.5	0.63	50.2	14.8	49.1
	1.1; 13 + 16	70.4	131.8	224.9	0.53	42.7	15.1	60.7

**Table 3:** List of wheat varieties used in this study with their respective *Glu-1* alleles encoding the HMW-GS.

Variety	*Glu-A1*		*Glu-B1*		*Glu-D1*	
		
	Allele	HMW-GS	Allele	HMW-GS	Allele	HMW-GS
‘Fukihokomugi’	b	2 *	b	7 + 8	f	2.2 + 12
‘Barbela 28’	*	1.1	f	13 + 16	a	2 + 12
‘PI 355’	*	*	*	*	*	*
‘Carala’	a	1	f	13 + 16	a	2 + 12
‘211.12014’	f	2··	c	7 + 9	d	5 + 10
‘13-21’	b	2*	b	7 + 8	d	5 + 10
‘Konini’	a	1	a	7	a	2 + 12
‘Atlas 66’	b	2*	f	13 + 16	a	2 + 12

* Not attributed.
